# Dechorionated zebrafish embryos improve evaluation of nanotoxicity

**DOI:** 10.3389/ftox.2024.1476110

**Published:** 2024-11-07

**Authors:** Rosa Kim, Yunwi Heo, Hakwon Yoon, June-Woo Park

**Affiliations:** ^1^ Environmental Exposure and Toxicology Research Center, Korea Institute of Toxicology (KIT), Jinju, Republic of Korea; ^2^ Department of Ocean Integrated Science, Chonnam National University, Yeosu, Republic of Korea; ^3^ College of Veterinary Medicine, Gyeongsang National University, Jinju, Republic of Korea; ^4^ Department of Biological Environment, Kangwon National University, Chuncheon, Republic of Korea; ^5^ Human and Environmental Toxicology Program, Korea University of Science and Technology (UST), Daejeon, Republic of Korea

**Keywords:** nanomaterials, zebrafish embryo, dechorionation, fish embryo toxicity, acute toxicity

## Abstract

**Introduction:**

In response to the growing need to evaluate nanomaterial (NM) toxicity and compliance with the “3Rs” principles (replacement, reduction, and refinement of animal experiments), zebrafish (*Danio rerio*) embryos have emerged as a promising alternative model for studies on NM toxicity. However, zebrafish embryos are surrounded by an acellular envelope, the chorion, which limits the permeability of NMs. The present study investigated the importance of dechorionated zebrafish embryos for evaluating NM toxicity.

**Methods:**

We utilized confocal microscopy and field-emission scanning electron microscopy with energy-dispersive spectroscopy to observe the permeability of NMs into the embryonic body using 50-nm fluorescein 5 (6)-isothiocyanate-incorporated silica nanoparticles (FITC-SiO_2_NPs). We investigated the physiological effects of removing the chorion using pronase on zebrafish embryos. Nanotoxicity was compared depending on the presence or absence of the chorion in zebrafish embryos using the standardized method ISO/TS 22082:2020.

**Results:**

The FITC-SiO_2_NPs were adsorbed onto the embryonic chorion; the Si content was higher in the chorion than in the embryonic body and higher in the intact zebrafish embryos than in the dechorionated ones. Dechorionated zebrafish embryos exhibited no negative physiological effects. The LC_50_ values of several NMs were lower in dechorionated embryos than those in intact ones.

**Conclusion:**

Dechorionated zebrafish embryos exhibited greater sensitivity to NMs than usual. To the best of our knowledge, this is the first study to evaluate NM toxicity using a new standardized test method, ISO/TS 22082:2020, and could contribute towards the increased utility of dechorionated embryos as an alternative model for the evaluation of nanotoxicity.

## 1 Introduction

Advances in nanotechnology have led to the increased production and extensive use of nanomaterials (NMs) in various applications, including consumer products and pharmaceuticals, thereby increasing the potential for exposure of humans and the environment to NMs (Giese et al., 2018). To ensure the safety of NMs, their toxicity must be precisely identified. However, conventional test guidelines, originally developed for evaluating the toxicity of water-soluble chemical substances, pose challenges when applied to NMs owing to their distinctive thermodynamic properties, including their size, shape, surface area, and surface charge. The Organisation of Economic Co-operation and Development (OECD), the United States Environmental Protection Agency, and the European Chemicals Agency have all amended conventional test guidelines through several projects, such as NANOMET, the National Nanotechnology Initiative, and NanoHarmony, respectively. Recently, as part of these efforts, a guidance document on the toxicological testing of aquatic and sediment samples for NMs has been published (OECD, 2020). However, the document is generally broad, necessitating the development of clear, pragmatic, and validated protocols to ensure the relevance and reliability of the results obtained.

Additionally, the International Specification Organization (ISO) has published technical specifications (TS) regarding the toxicity of NMs by Technical Committee 229/Working Group 3 as follows: ISO/TS 5387:2023, ISO/TS 20787:2017, ISO/TS 23034:2021, and ISO/TS 21633:2021. These specifications, which rely on *in vitro* assays or animal testing, have the following limitations: *in vitro* assay may not account for the inherent complexity of organ systems, and animal testing is time-consuming, expensive, and has ethical issues related to animal use. Therefore, a standardized method based on non-animal use that is cost-efficient and suitable for the large-scale screening of nanotoxicity is needed.

The fish embryo toxicity test is an assay specified by the OECD for hazard classification and risk assessment of potentially harmful substances (Busquet et al., 2014). Zebrafish (*Danio rerio*), commonly used for fish embryo toxicity test, have several characteristics, including their small size, ease of maintenance under laboratory conditions, high embryo production, and short embryogenesis, which allows for high-throughput testing (Pereira et al., 2019). Moreover, zebrafish embryos are regarded as an alternative to other animal testing models because of their high genetic, anatomical, and physiological similarity to humans and compliance with the 3Rs principles (replacement, reduction, and refinement of animal experiments), as outlined in regulatory frameworks, which allow for the translation of toxicity information to other vertebrates (Busquet et al., 2014; de Jong et al., 2011). Therefore, zebrafish embryos are widely used to evaluate the toxicity of potentially hazardous substances.

Zebrafish embryos offer notable experimental flexibility and versatility for nanotoxicity studies (Pereira et al., 2019). OECD Test Guideline No. 236, which is still widely used to evaluate the toxicity of NMs, recommends that other suitable test methods should be applied for substances with high molecular weights (i.e., ≥3 kDa) and a very bulky molecular structure. This means that the chorion may confound evaluations of NM toxicity. The chorion of zebrafish embryos is 1.5–2.5 μm thick, consists of three layers pierced by the chorionic pore canals (CPCs), and has evenly distributed pores (diameter, 0.5–0.7 μm) that are present 1.5–3 μm apart (Bonsignorio et al., 1996; Laale, 1977; Rawson et al., 2000), protecting against physical or chemical stimuli. A few studies have shown that chorion limits the permeability of NMs. For example, 60- or 200-nm silica nanoparticles (SiO_2_NP) adhered to the chorion of zebrafish embryos and exhibited no uptake or translocation (Fent et al., 2010), and single-walled carbon nanotubes adhered to the chorion of zebrafish embryos (Cheng et al., 2007). Therefore, removing the chorion of zebrafish embryos should be considered a means of improving NM toxicity evaluations.

The International Specification Organization (ISO) published the standardized test method for evaluating nanotoxicity using dechorionated zebrafish embryos (ISO/TS 22082:2020). It introduced an optimized method for the dechorionation of zebrafish embryos using pronase (a protease from *Streptomyces griseus*) and detailed how to conduct a nanotoxicity assay on these embryos. However, removing the chorion may damage the embryos themselves, resulting in high mortality (Henn and Braunbeck, 2011; Mandrell et al., 2012). Therefore, dechorionation should be performed using a standardized method, considering the physiological effects of the dechorionation.

The present study aimed to determine the importance of chorion removal from zebrafish embryos for reproducible nanotoxicity evaluation by comparing nanotoxicity between intact (i.e., chorionated) and dechorionated zebrafish embryos, which were obtained as proposed in a new standardized method, ISO/TS 22082:2020. We hypothesize that the difference in nanotoxicity between intact and dechorionated zebrafish embryos could be attributed to the chorion blocking the NMs from directly making contact with the embryonic body. Therefore, our study investigates the transport of NMs by the chorion into the embryonic body and the physiological effects of dechorionated zebrafish embryos. Furthermore, we compare the nanotoxicity in intact and dechorionated zebrafish embryos. A few studies evaluated the nanotoxicity using dechorionated embryos, however, these have been performed using non-standardized methods, which may result in low reproducibility (Carbaugh et al., 2022; Paatero et al., 2017). Our study looks forward to increasing the utilization of dechorionated embryos as an alternative model for evaluating nanotoxicity.

## 2 Materials and methods

### 2.1 NMs

Fluorescein 5 (6)-isothiocyanate-incorporated SiO_2_NPs (FITC-SiO_2_NPs) with a diameter of 50 nm were used to determine the restricted uptake of NMs by the chorion; these were kindly provided by the Korea Research Institute of Standards and Science (Daejeon, Korea). The NMs used to compare the mortality of zebrafish embryos in the presence and absence of the chorion were as follows (Table 1): non-coated silver nanoparticles with a diameter of 30 nm (30AgNPs-B); polypyrrolidone (PVP)-coated AgNPs with a diameter of 5 nm (5AgNPs-P); 10ZnONPs-B, non-coated zinc oxide nanoparticles (ZnONPs) with a diameter of 10–30 nm (10ZnONPs-B); PVP-coated multi-walled carbon nanotubes with an outer diameter of 7 nm (7MWCNTs-P); and PVP-coated MWCNTs with an outer diameter of 50 nm (50MWCNTs-P). Physicochemical information supplied from the manufacturer has been summarized in Supplemental Table S1. The hydrodynamic size and polydispersity index (PDI) of each NM in the test medium were measured using a ZetaSizer Nano (Nano ZS 90, Malvern Instrument, Worcestershire, UK). The hydrodynamic size of each tested NM was not significantly changed over 24 h (Table 1).

**TABLE 1:** Characterization of the tested nanomaterials in the test medium.

Type	Hydrodynamic size (nm)		Polydisperse index	
	0 h	24 h	0 h	24 h
30AgNPs-B	85.22 ± 1.28	87.69 ± 2.96	0.35 ± 0.03	0.39 ± 0.02
5AgNPs-P	15.87 ± 0.35	13.57 ± 0.17	0.38 ± 0.01	0.30 ± 0.01
10ZnONPs-B	248.10 ± 4.58	253.92 ± 4.66	0.24 ± 0.05	0.27 ± 0.05
7MWCNTs-P	295.57 ± 26.67	290.6 ± 6.99	0.55 ± 0.03	0.53 ± 0.05
50MWCNTs-P	202.70 ± 2.39	204.17 ± 3.16	0.25 ± 0.01	0.25 ± 0.01

30AgNPs-B, non-coated silver nanoparticles with a diameter of 30 nm; 5AgNPs-P, polypyrrolidone (PVP)-coated AgNPs, with a diameter of 5 nm; 10ZnONPs-B, non-coated zinc oxide nanoparticles with a diameter of 10–30 nm; 7MWCNTs-P, PVP-coated multi-walled carbon nanotubes with an outer diameter of 7 nm; 50MWCNTs-P, PVP-coated MWCNTs, with an outer diameter of 50 nm.

### 2.2 Zebrafish embryos

Wild-type zebrafish (*D. rerio*; AB) were reared at the Korea Institute of Toxicology (Jinju, Korea) under a 16:8 h light:dark cycle and at a constant temperature of 27°C ± 1°C in carbon-filtered dechlorinated tap water (embryo medium). The zebrafish were fed freshly hatched brine shrimp (*Artemia nauplii*) (Salt Lake City, UT, United States) or commercial food flakes (TetraMin; Tetra Werke, Melle, Germany), divided into two daily feedings. To obtain embryos, sexually mature male and female zebrafish (1:1 ratio) were placed in a light-blocked box overnight before spawning stimulation. The following morning, the lights were turned on to stimulate spawning. Fully fertilized, healthy embryos were collected within 1 h in a Petri dish filled with embryo medium. Zebrafish embryos were kept at 27°C ± 1°C until the experiment. This study involving animals was reviewed and approved (No. 2110-0005) by the Institutional Animal Care and Use Committee of Korea Institute of Toxicology (KIT, Jinju, Korea).

### 2.3 Transport of NMs into zebrafish embryos

Two independent experiments were performed to determine the limited transport of NMs by the chorion into zebrafish embryos. In the first series of experiments, the chorion and perivitelline space (between the chorion and the embryo) of intact zebrafish embryos (with chorion) exposed to FITC-SiO_2_NPs were visually observed using a confocal microscope (Nikon A1R; Nikon Corporation, Melville, NY, United States). Intact zebrafish embryos at 6 h post-fertilization (hpf) were exposed to FITC-SiO_2_NPs for 48 h in a 6-well plate, with each well containing 2 mL of the solution under the same breeding conditions. Sixty embryos (10 embryos per well) were used in each test group. The exposure concentration of FITC-SiO_2_NPs was set to 10 mg/L based on preliminary testing, as this concentration showed no effect on mortality and malformations. At the end of exposure, intact zebrafish embryos were anesthetized using 0.1 g/L tricaine methanesulfonate (MS-222; Cas No. 886-86–2, Sigma-Aldrich, St. Louis, MO, United States) and rinsed several times with deionized water. All embryos were observed under a confocal microscope (Nikon A1R).

The second series of experiments was conducted to measure the Si content in the chorion and/or embryonic body to confirm the presence of NMs using a field-emission scanning electron microscope (FE-SEM) equipped with energy-dispersive spectroscopy (EDS) apparatus (Sirion; FEI, Eindhoven, the Netherlands). Intact and dechorionated zebrafish embryos were exposed to 10 mg/L FITC-SiO_2_NPs under the same conditions as in the first experiment. At the end of exposure, the intact and dechorionated zebrafish embryos were rinsed with deionized water followed by fixation with 4% paraformaldehyde overnight at 4°C. Subsequently, the chorion or body of intact zebrafish embryos was obtained by manual dechorionation.

### 2.4 Dechorionation

#### 2.4.1 Dechorionation protocol

The chorion was removed according to the procedure outlined in ISO/TS 22082:2020 (ISO, 2020). Intact zebrafish embryos were treated with 0.764 U/mL pronase (protease from *S. griseus*; CAS No. 9036-06–0, Sigma-Aldrich) at 4 hpf for 5 min in a glass dish and then rinsed with embryo medium (pronase-free water). After rinsing, the zebrafish embryos were left to rest for 20–30 min, followed by rinsing at least three times. Healthy dechorionated zebrafish embryos were used for NM exposure.

#### 2.4.2 Physiological effects of dechorionation on zebrafish embryos

This study investigated the physiological effects of pronase-mediated dechorionation on the survival, heartbeat, and behavioral changes (total distance moved and velocity) of zebrafish embryos. The test group consisted of intact and dechorionated zebrafish embryos, which were compared with a positive control group and with each other. Chemicals used for the positive control differed according to each endpoint as follows: 4 mg/L 3,4-dichloroaniline (3,4-DCA; CAS No. 95-76-1, Sigma-Aldrich) for the evaluation of lethality, as proposed in OECD TG 236 (Adeyemi et al., 2015; OECD, 2013); 458.57 μg/L astemizole (CAS No. 68844-77-9, Sigma-Aldrich), which is recognized as a ventricular arrhythmia inducer that inhibits the heartbeat in zebrafish (Langheinrich et al., 2003), leading to cardiotoxicity, for the evaluation of cardiotoxicity; and 138.17 mg/L pentylenetetrazole (PTZ; CAS No. 92-84-2, Sigma-Aldrich), which is a common convulsant that induces seizure-like behavior (Baraban et al., 2005), for evaluating locomotor activity.

Each test was performed in triplicate. All test organisms were exposed from 6 to 120 hpf. The 6-well plate was used as the test vessel. Thirty embryos (ten embryos per well) were used in each test group. The zebrafish embryos were maintained under the same breeding conditions. Embryos were considered dead if they exhibited coagulation, lack of somite formation, non-detachment of the tail, or lack of heartbeat, as observed under a stereomicroscope (SMZ1000; Nikon, Japan) (OECD, 2013). The heartbeat of zebrafish embryos was measured for 20 s under a stereomicroscope after anesthetization using 0.1 g/L MS-222 and was expressed in beats per minute. For locomotor analysis, zebrafish embryos were first transferred to a 48-well plate with one larva per well in 400 μL of solution. The 48-well plate was placed in an observation chamber (Noldus, Wageningen, the Netherlands) and acclimated for 10 min in the dark. Behavior was recorded for 10 min under light conditions (Irons et al., 2010). Measurements (total distance moved and velocity) were performed for each zebrafish embryo using an EthoVision XT 11 image analysis system (Noldus).

### 2.5 Zebrafish embryo nanotoxicity assay

**FIGURE 1:**
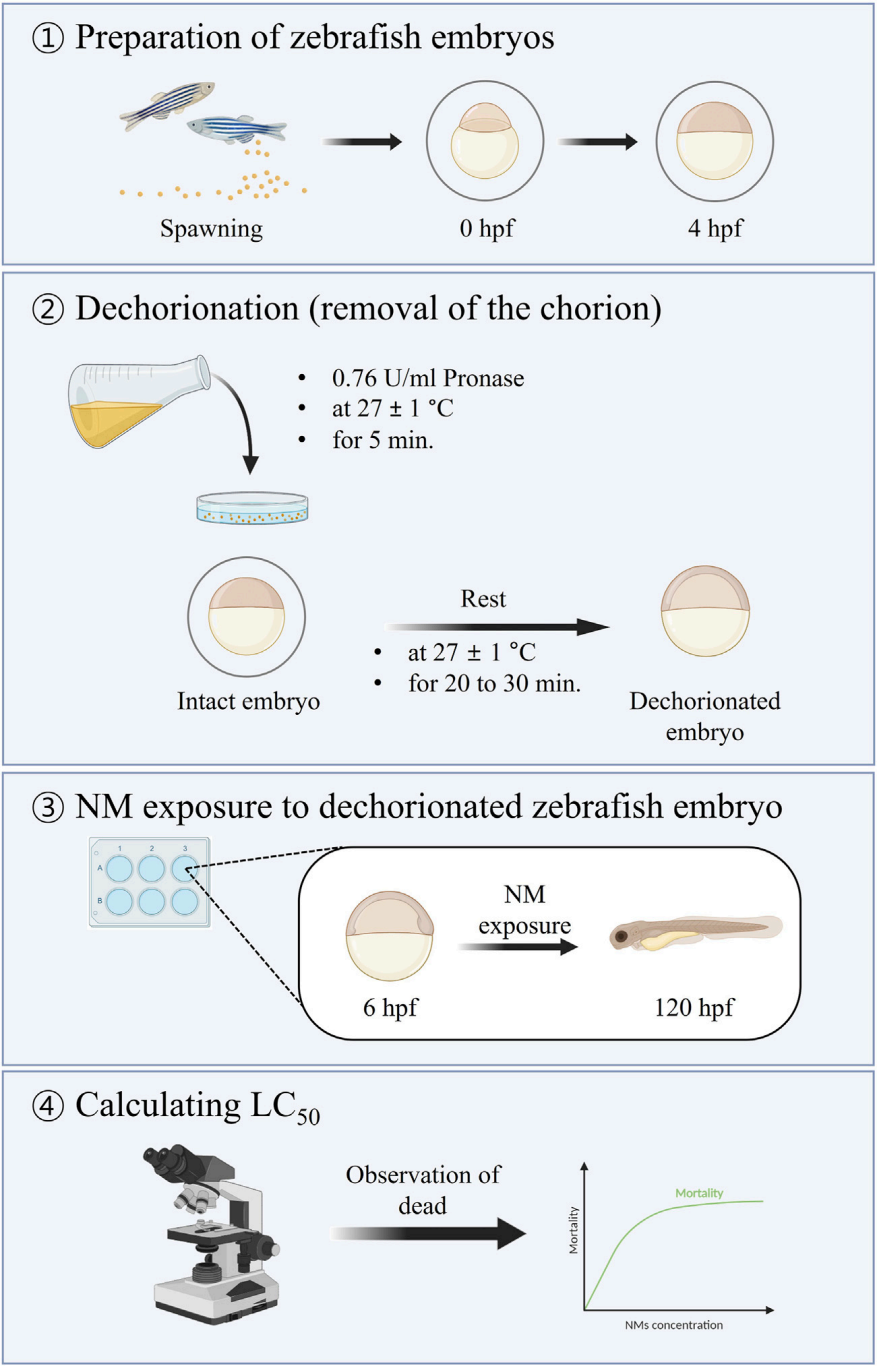
Summary of the method for assessing nanotoxicity using dechorionated zebrafish embryos (ISO, 2020).

Nanotoxicity in intact (with chorion) or dechorionated (without chorion) zebrafish embryos was assessed according to the ISO/TS 22082:2020 guidelines (ISO, 2020). The procedure used in this study is illustrated in Figure 1.

#### 2.5.1 Preparation of zebrafish embryos

The collected embryos, as described in Section 2.2, were placed in a Petri dish with carbon-filtered dechlorinated tap water until the experiment.

#### 2.5.2 Dechorionation

Dechorionation was conducted according to the method outlined in Section 2.4.1. This procedure was exclusively used to evaluate nanotoxicity in dechorionated zebrafish embryos.

#### 2.5.3 NM exposure

The stock solution of NMs (1,000 mg/L) in deionized water was sonicated for 30 min with a continuous output of 35 W using a probe-type sonicator (Sonics, Sonics and Materials Inc., Newtown, CT, United States). The treatment solutions of NMs were prepared by serially diluting the stock solution in embryo medium, and their concentrations were as follows: 0 (0.01% bovine serum albumin, BSA), 1.6, 3.1, 6.3, 12.5, 25, 50, and 100 mg/L for 30AgNPs-B and 5AgNPs-P and 0 (0.01% BSA), 0.1, 1, 10, and 100 mg/L for 7MWCNTs-P and 50MWCNTs-P. The solubilizing agent was 0.01% BSA, which was the same for all exposure solutions. The treatment solution was thoroughly renewed every 24 h until the end of the experiment. Intact or dechorionated zebrafish embryos were exposed to NMs from 6 to 120 hpf in 6-well plates. Temperature and photoperiod were maintained at 27°C ± 1°C with a 16:8 h light/dark cycle during exposure.

#### 2.5.4 Observation of dead embryos

Embryos were considered dead when any of the following were observed: coagulation of embryos; lack of somite formation; non-detachment of the tail; and lack of heartbeat. Dead organisms were immediately removed from the test vessel.

#### 2.5.5 Calculation of LC_50_

LC_50_ (lethal concentration that caused the death of 50% of the test organisms) with 95% confidence intervals was calculated using the Comprehensive Environmental Toxicity Information System (Tidepool Scientific Software, McKinleyville, CA, United States).

### 2.6 Statistical analysis

All data were analyzed using SPSS Statistics software (version 25.0; IBM, Armonk, NY, United States). The normality of the data distribution was analyzed using the Shapiro–Wilk test, and homogeneity of variance was evaluated using Levene’s test. For mortality and locomotor activity (total distance moved and velocity), the data were not normally distributed; thus, the statistical significance between test groups was evaluated using the Kruskal–Wallis test, followed by the Mann–Whitney *U* test. For heartbeats, the data were normally distributed but did not satisfy the homogeneity of variance assumption. Therefore, Dunnett’s T3 test was used to determine the statistical significance between test groups. Differences were considered statistically significant at *p* < 0.05.

## 3 Results

### 3.1 Transport of NMs into zebrafish embryos

**FIGURE 2:**
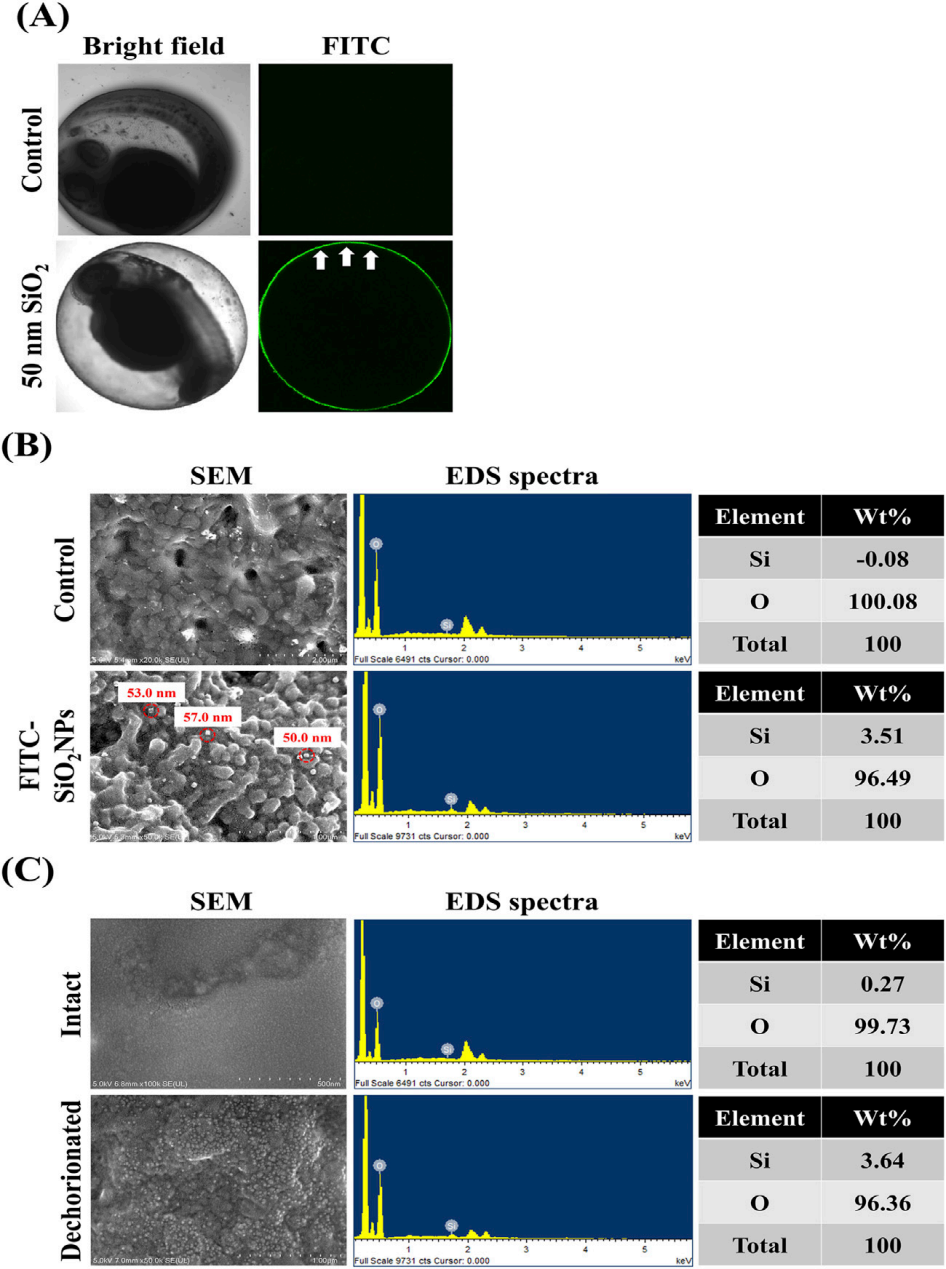
Limited transport of nanomaterials by the chorion of zebrafish. **(A)** Confocal microscopic images of the zebrafish embryos exposed to fluorescein 5 (6)-isothiocyanate-incorporated silica nanoparticles (FITC-SiO_2_NPs) with a 50-nm diameter. **(B)** Field-emission scanning electron microscope (FE-SEM) images and energy-dispersive spectroscopy (EDS) spectra of the chorion of FITC-SiO_2_NPs-exposed zebrafish embryos. **(C)** SEM images and EDS spectra of the body of intact or dechorionated zebrafish embryos exposed to FITC-SiO_2_NPs. When intact zebrafish embryos were exposed to FITC-SiO_2_NPs, they were manually dechorionated at the end of the exposure period.

This study investigated whether the transport of NMs into zebrafish embryos is restricted by the chorion. Figure 2A shows the visual inspection of FITC-SiO_2_NPs -treated zebrafish embryos using confocal microscopy. Untreated embryos exhibited no green fluorescence in the chorion and perivitelline space (between the chorion and the embryonic body). The FITC-SiO_2_NPs-treated embryos exhibited green fluorescence on the chorion surface with no green fluorescence observed in the perivitelline space, distinctly discernible from the autofluorescence observed in some tissues (such as the retina and yolk sac) in the non-treated and FITC-SiO_2_NPs-treated embryos. Figure 2B shows FE-SEM images and EDS spectra of the chorion of untreated and FITC-SiO_2_NPs -treated embryos. Spherical particles with diameters of 50.0–57.0 nm were detected at the chorion in the FITC-SiO_2_NPs -treated embryos, confirmed to contain Si (3.51 wt%) by EDS spectra, whereas the Si signal was not detectable in the non-treated embryos. This result showed that the green fluorescence observed in the chorion of FITC-SiO_2_NPs-treated embryos could be attributed to the presence of silica nanoparticles. However, this does not mean that the embryos take up NMs through the chorion, as confocal microscopy may have limitations in the detection of NMs, therefore, intact and dechorionated embryos exposed to FITC-SiO_2_NPs were analyzed using FE-SEM images and EDS spectra (Figure 2C). Spherical particles were more visible in dechorionated embryonic bodies than in intact ones. Additionally, the weight of Si was 0.27% and 3.64% in intact and dechorionated embryos, respectively.

### 3.2 Physiological effects of dechorionation in zebrafish embryos

**FIGURE 3:**
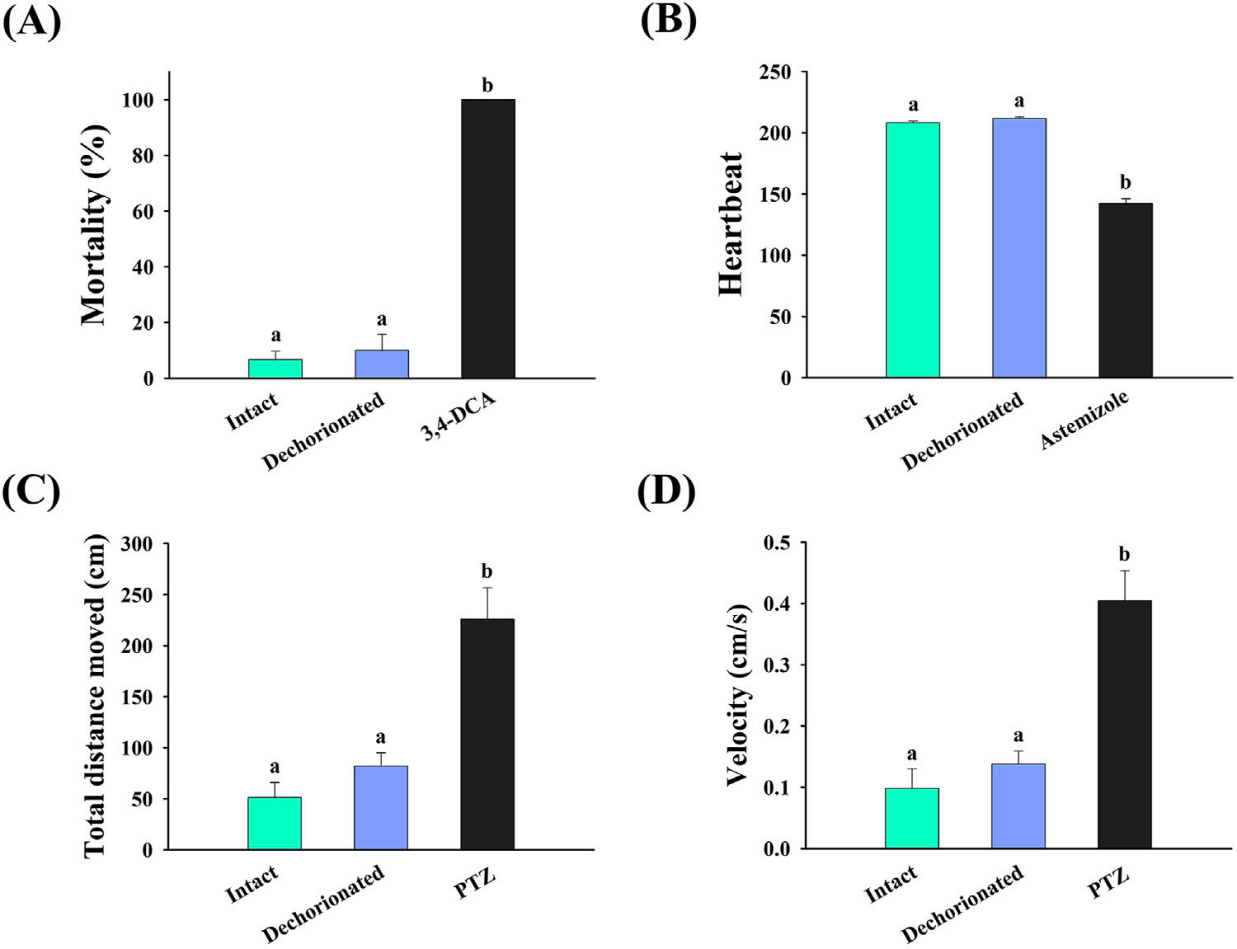
Effects of chorion removal using pronase on the **(A)** survival, **(B)** heartbeat, **(C)** total distance moved, and **(D)** velocity of zebrafish embryos. Different letters represent significant differences between treatments (Mann–Whitney *U* test or Dunnett T3, *p* < 0.05). PTZ, pentylenetetrazole; 3,4-DCA, 3,4-dichloroaniline.

The physiological effects of dechorionation on zebrafish embryos are shown in Figure 3. The mortality rates of the intact and dechorionated embryos were 6.67% ± 3.04% and 10.00% ± 5.77%, respectively, with no significant difference (*p* > 0.05) (Figure 3A), whereas intact embryos exposed to 4 mg/L 3,4-DCA died (Figure 3A). In terms of heartbeat, no difference was observed between the intact and dechorionated zebrafish embryos (*p* < 0.05), whereas the heartbeat in the intact embryos exposed to 458.57 μg/L astemizole was significantly decreased (*p* < 0.05). Regarding locomotor activity, no difference was observed in total distance moved and velocity between intact and dechorionated embryos (*p* > 0.05), whereas 138.17 mg/L PTZ induced hyperactivity in intact embryos (*p* < 0.05).

### 3.3 Comparison of nanotoxicity in intact and dechorionated embryos

**FIGURE 4:**
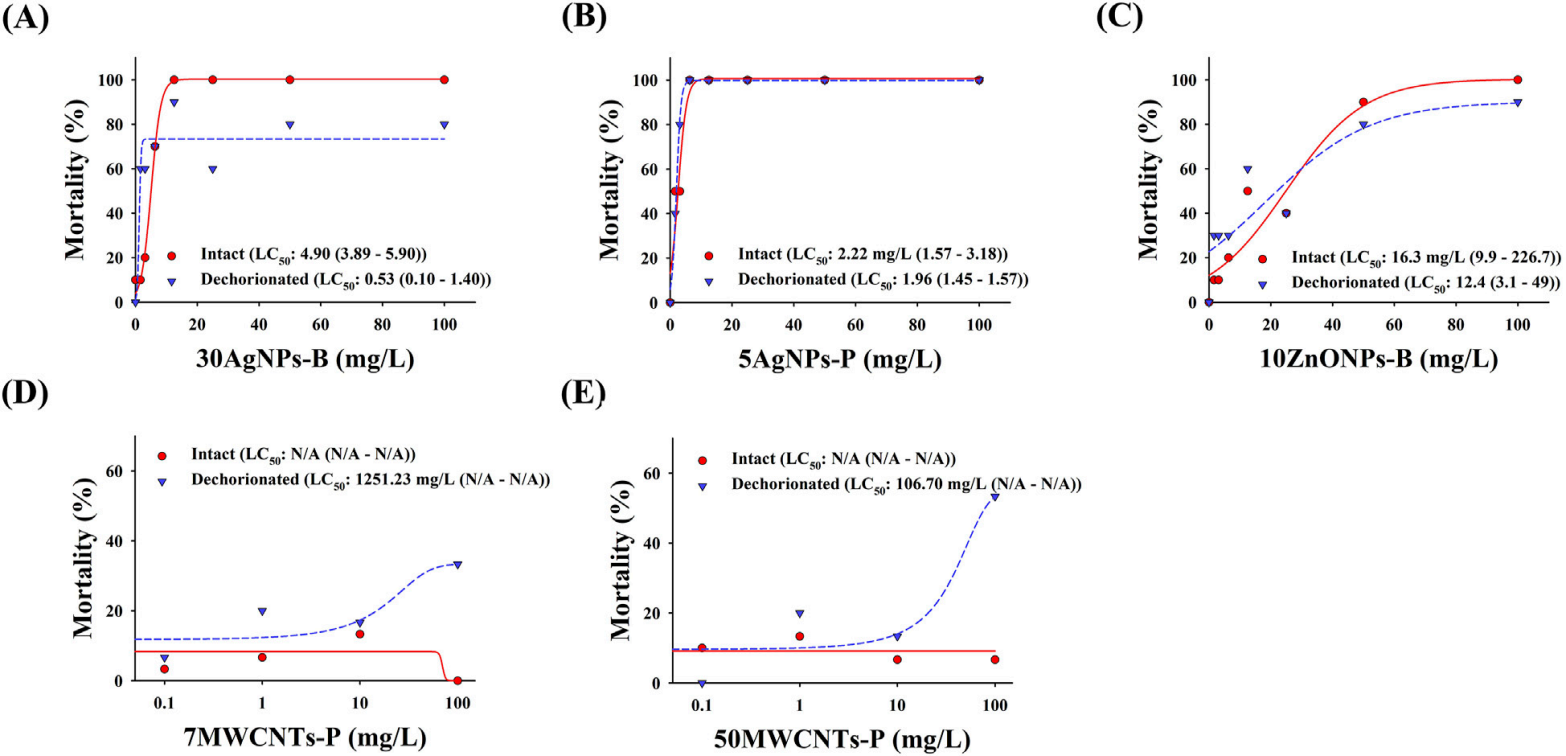
Mortality in the intact and dechorionated zebrafish embryos exposed to various nanomaterials. **(A)** 30AgNPs-B, 30-nm non-coated silver nanoparticles (AgNPs); **(B)** 5AgNPs-P, 5-nm polypyrrolidone (PVP)-coated AgNPs; **(C)** 10ZnONPs-B, 10–30-nm non-coated zinc oxide nanoparticles (ZnONPs); **(D)** 7MWCNTs-P, 7-nm PVP-coated multi-walled carbon nanotubes (MWCNTs); **(E)** 50MWCNTs-P, 50-nm PVP-coated MWCNTs.

The mortality of zebrafish embryos with and without chorions is shown in Figure 4. The LC_50_ of 30AgNPs-B for intact and dechorionated embryos were 4.90 mg/L and 0.53 mg/L, respectively. The LC_50_ of 5AgNPs-P for the intact and dechorionated embryos was 2.22 mg/L and 1.96 mg/L, respectively. The LC_50_ of 10ZnONPs-B for intact and dechorionated embryos were 16.3 mg/L and 12.4 mg/L, respectively. The LC_50_ of 7MWCNTs-P was not determined for the intact embryos, whereas it was 1,251.23 mg/L for the dechorionated embryos. The LC_50_ of 50MWCNTs-P was not calculated for intact embryos, whereas it was 106.70 mg/L for dechorionated embryos.

## 4 Discussion

### 4.1 Limited transport of NMs into zebrafish embryos

This study investigated the transport of NMs into the embryonic bodies using confocal microscopy, FE-SEM, and EDS analyses. Our results showed that NMs adhered to the chorion, implying that they can become trapped inside CPCs, inhibiting their transport to the embryonic body. This assumption is supported by the higher Si content in the chorion compared with that in the embryonic body, as shown in this study, indicating that some nanomaterials could contact with the embryonic bodies. A study on the quantification of NPs in intact zebrafish embryos exposed to AgNPs, gold nanoparticles (AuNPs), and aluminum oxide nanoparticles (Al_2_O_3_NPs) at 24 hpf using laser ablation coupled with inductively coupled plasma mass spectrometry showed that NP accumulation in the chorion was markedly higher than that in the embryonic bodies (Böhme et al., 2015). Another study showed that 40-nm SiO_2_NPs were more abundant on the chorion of zebrafish embryos than on the embryonic body, and that this tendency increased with prolonged exposure (Chao et al., 2018). Assuming that the pore diameter of zebrafish embryos is 0.5–0.7 μm, NMs can pass through the chorion. However, most NMs cannot pass through the chorion (Böhme et al., 2015; Chao et al., 2018; Fent et al., 2010; Lee et al., 2007). This could be attributed to NMs becoming trapped in the chorion, where they can aggregate with other incoming NMs, blocking the CPCs (Chao et al., 2018; Lee et al., 2007). Collectively, most NMs could remain the chorion, with only a small amount of NMs could enter into the embryonic body. Therefore, the chorion of zebrafish embryos could trap NMs inside the CPCs, thereby serving as a biological barrier.

### 4.2 Physiological effects of dechorionation in zebrafish embryos

ISO/TS 22082:2020 highlights the importance of the removal of chorion when evaluating using zebrafish embryos (ISO, 2020). The zebrafish chorion can be removed through two approaches: mechanical removal using forceps and enzymatic treatment with pronase; the latter of which offers several advantages over the mechanical dechorionation method, such as time and labor efficiency due to easier preparation for dechorionation and the ability to obtain a large number of dechorionated embryos. Although pronase treatment is widely used to dechorionate zebrafish embryos (Westerfield, 2007), there is a concern that pronase treatment may have negative effects on organisms (Henn and Braufnbeck, 2011; Mandrell et al., 2012). The survival rate of embryos dechorionated with 4 × 10^−3^ U/mL pronase at 6 hpf was 20% (Henn and Braunbeck, 2011), indicating that the pronase treatment was mostly detrimental to 6 hpf embryos. Our demonstration of pronase-supported removal of the chorion, at 4 hpf yielded about 90% survival and maintained normal physiological conditions up to 120 hpf, indicating that the developmental stage may need to be considered during pronase-mediated dechorionation. Mortality, heartbeat, and locomotor activity provide a comprehensive understanding of the physiological responses of aquatic organisms to stress (Fraysse et al., 2006). Our study showed that the mortality, heartbeat, and locomotor activity of dechorionated embryos were similar to those of intact embryos, implying that dechorionation using pronase treatment has no adverse effects on the physiological condition of zebrafish embryos. Therefore, the pronase-supported approach described in ISO/TS 22082:2020 can be reliable and practical for the evaluation of nanotoxicity.

### 4.3 Difference in nanotoxicity between intact and dechorionated embryos

Based on the limited transport of NMs into the embryonic body and integrity of the dechorionated zebrafish embryos, we compared the nanotoxicity between intact and dechorionated zebrafish embryos using the standardized method ISO/TS 22082:2020. Our results show that NM toxicity was higher in dechorionated embryos than in intact ones, suggesting that dechorionated embryos better reflect NM toxicity. Similarly, previous studies showed that the mortality of 20-nm and 110-nm AgNPs and 25-nm SiO_2_NPs was higher in the dechorionated embryos than in the intact ones (Kim and Tanguay, 2014; Vranic et al., 2019). Nanotoxicity has been suggested to be induced by the nanomaterials themselves or the ions released from them (Beer et al., 2012; Gomes et al., 2013; Zhou et al., 2014). This study did not measure released ions or anything else derived from the NMs; therefore, it is difficult to ascertain whether any NM-induced toxicity originated from NMs themselves and/or the released ions. Nevertheless, it is noteworthy that dechorionated embryos exhibited higher toxicity compared to intact embryos. The higher mortality seen in dechorionated embryos could be attributed to the removal of the chorion, allowing increased direct contact between the nanoparticles and the embryonic bodies. Additionally, the epidermis could be the route of entry for internalization of nanoparticles while the embryo is not actively feeding. Nanoparticles could be internalized by causing local injuries to the fragile epidermis of the embryos (Asharani et al., 2008; van Pomeren et al., 2017; Zhu et al., 2008). Notably, during the early embryonic stages, the nanoparticles internalized through the epidermis have been shown to be evenly distributed throughout the embryonic bodies (Asharani et al., 2008; Asharani et al., 2011; Browning et al., 2009), implying the importance of direct exposure to nanomaterials from the early embryonic stage. However, due to the limited transport of most nanoparticles by the chorion into the embryonic bodies, as previously mentioned, intact embryos could be practically exposed to nanomaterials after hatching. Therefore, dechorionated embryos can have increased contact with NMs, potentially making them more susceptible to nanotoxicity.

Furthermore, NMs trapped inside CPCs or adhering to the chorion surface may generate a hypoxic microenvironment (Cordeiro et al., 2018) that could negatively affect the early development of zebrafish embryos (Ong et al., 2014), thereby hindering the accurate assessment of inherent nanotoxicity. Despite the removal of the chorion being environmentally irrelevant, test methods involving the use of dechorionated zebrafish embryos could enhance contact with NMs and exclude side effects resulting from NMs trapped in the chorion. Therefore, our study suggests that dechorionated embryos can sensitively reflect nanotoxicity without the unwanted effects attributed to chorion removal.

## 5 Conclusion

In this study, we confirmed that the evaluation of nanotoxicity using dechorionated zebrafish embryos could be more appropriate than using intact zebrafish embryos in zebrafish toxicity test such as FET assay (OECD TG 236, Fish Embryo Acute Toxicity Test). However, it is important to investigate any unintentional impacts induced by dechorionating procedures and any potential toxic mechanisms in dechorionated zebrafish embryos; these should be explored.

## Data Availability

The raw data supporting the conclusions of this article will be made available by the authors, without undue reservation.
