# Supplementary material for: Dechorionated zebrafish embryos improve evaluation of nanotoxicity
Source: Front Toxicol. 2024 Nov 7;6:1476110. doi: 10.3389/ftox.2024.1476110 (PMC11578979; doi:10.3389/ftox.2024.1476110)
Supplement: Supplementary file 1 [file Table1.DOCX]

Supplementary Material

Dechorionated Zebrafish Embryos Improve Evaluation of Nanotoxicity

Rosa Kim^1,2^, Yunwi Heo^1,3^, Hakwon Yoon^4^, June-Woo Park ^1,5*^

^1^Environmental Exposure & Toxicology Research Center, Korea Institute of Toxicology (KIT), Jinju 52834, Republic of Korea

^2^Department of Ocean Integrated Science, Chonnam National University, Yeosu 59626, Republic of Korea

^3^College of Veterinary Medicine, Gyeongsang National University, Jinju, 52828, Republic of Korea

^4^Department of Biological Environment, Kangwon National University, Chuncheon 24341, Republic of Korea

^5^Human and Environmental Toxicology Program, Korea University of Science and Technology (UST), 217, Gajeong-ro, Daejeon 34113, Republic of Korea

*** Correspondence:**June-Woo Park, PhD
[jwpark@kitox.re.kr](mailto:jwpark@kitox.re.kr)

Tel: +82-55-750-3833; Fax: +82-55-750-3799

Number of Pages: 1

Number of Tables: 1

Running title: Dechorionated Embryos for Evaluating Nanotoxicity

**Table S1**. The physical properties of tested nanomaterials, provided by the manufacturer.

| Type | Diameter (nm) | Length (μm) | Coating material | Manufacturer |
| --- | --- | --- | --- | --- |
| 30AgNPs-B | 30 | None | None | NanoComposix |
| 5AgNPs-P | 5 | None | Polypyrrolidone | NanoComposix |
| 10ZnONPs-B | 10–30 | None | None | US Research Nanomaterials |
| 7MWCNTs-P | Outer: <7  Inner: 2–5 | 10–30 | Polypyrrolidone | US Research Nanomaterials |
| 50MWCNTs-P | Outer: 50–80  Inner: 5–15 | 10–20 | Polypyrrolidone | US Research Nanomaterials |

30AgNPs-B, non-coated silver nanoparticles with a diameter of 30 nm; 5AgNPs-P, polypyrrolidone (PVP)-coated AgNPs with a diameter of 5 nm; 10ZnONPs-B, non-coated zinc oxide nanoparticles with a diameter of 10 to 30 nm; 7MWCNTs-P, PVP-coated multi-walled carbon nanotubes with an outer diameter of 7 nm; 50MWCNTs-P, PVP-coated MWCNTs with an outer diameter of 50 nm
